# Atypical Presentation of Plasma Cell Neoplasm of the Sternum

**DOI:** 10.7759/cureus.16106

**Published:** 2021-07-02

**Authors:** Ndausung Udongwo, Steven Douedi, Mihir Odak, Abbas Alshami, Swapnil V Patel, Taliya Farooq

**Affiliations:** 1 Internal Medicine, Jersey Shore University Medical Center, Neptune City, USA; 2 Pathology, Jersey Shore University Medical Center, Neptune City, USA

**Keywords:** chest mass, multiple myeloma, plasma cell neoplasm, dutcher bodies, extramedullary myeloma

## Abstract

Multiple myeloma (MM) is a rare plasma cell neoplasm characterized by monoclonal cell infiltration in the bone marrow, which can cause anemia, bone pain, and recurrent infections. Extramedullary myeloma (EM) is a rare clinical presentation with a poor prognosis. It involves the accumulation of clonal plasma cells in soft tissues with a tumor-like appearance, either presenting as a primary (initial) or secondary (relapse) malignancy. We present a case of a 65-year-old male who experienced an abrupt onset of chest pain associated with a localized sternal mass while exercising the day prior to arrival. Chest computed tomography (CT) scan with contrast revealed an expansile lytic lesion around the sternal area. Due to high suspicion for malignancy, a CT-guided core needle biopsy was done, which showed plasma cells with rare Dutcher bodies consistent with MM. Bone marrow smear showed the presence of 70% plasma cells confirming a diagnosis of MM. Early detection of this devastating disease may help improve survival. Therefore, physicians should have a high index of suspicion for MM in older patients with similar clinical presentations.

## Introduction

Multiple myeloma (MM) is a rare plasma cell malignancy characterized by the abnormal production of monoclonal cells in the bone marrow. It accounts for about 1% of all cancers worldwide, and 10% of all blood cancers [1]. Its incidence worldwide is about 0.006%-0.007% per year [1]. The median age of onset is 65 years old and is twice as common in Blacks when compared to Caucasians [1]. It has variable clinical presentations, but most commonly causes kidney failure, hypercalcemia, anemia, recurrent infections, and bone pain. The diagnosis is confirmed by the presence of ≥10% of monoclonal plasma cells in the bone marrow or end-organ damage (symptomatic MM). Usually, plasma cells are confined in the bone marrow in patients with MM, but extramedullary infestations presenting as a mass have also been reported in the medical literature [2].

While the etiology of extramedullary myeloma (EM) is unknown, an initial presentation is rare and therefore a detailed physical examination, imaging studies, laboratory results, and core-needle/bone marrow biopsy are required to make an accurate diagnosis [2]. We present a case of a 65-year-old male who presented to the emergency department (ED) for an evaluation of a chest mass after an episode of chest pain. A core needle biopsy revealed histology findings consistent with MM, which was confirmed with a bone marrow biopsy 13 days later. To our knowledge, there are only a few reported cases of this unusual presentation.

## Case presentation

A 65-year-old male with a medical history of hypertension and hyperlipidemia presented to the ED to evaluate a chest mass, which he noticed while exercising the day prior. He initially experienced an abrupt, sharp localized pain in his anterior neck and chest area, which lasted for a few seconds before resolving spontaneously. He had a 7-pound (lbs) weight loss over the last two months, which he attributed to his increased exercise regimen. He denied any other complaints including fever, fatigue, night sweats, cough, shortness of breath, abdominal pain, or recent trauma. He denied any tobacco or illicit substance use and only drank alcohol occasionally. Family history was remarkable for prostate cancer in his father and unspecified cancers in his mother. His home medications were atorvastatin 10 milligrams (mg), amlodipine 10 mg, losartan 100 mg, and chlorthalidone 25 mg all taken once daily.

On physical examination, vitals were a blood pressure of 128/89 mmHg, heart rate of 87 beats per minute, respiratory rate of 18 breaths per minute, the temperature of 98.5 °F, and oxygen saturation of 100% on room air. He was in no acute distress on presentation. Physical exam was remarkable for a non-mobile hard mass measuring about 4 x 3 cm on the sternum and extending to the clavicles. No skin changes, erythema, rashes, or tenderness were appreciated. The cardiopulmonary examination was unremarkable on admission.

The laboratory results are shown in Table 1.

**Table 1:** Laboratory results

Blood	Results	Reference range
Hemoglobin (g/dL)	11.8	12.0–17.5
White blood cell count (10*3U/L)	5.6	4.5–11.0
Glucose (mg/dL)	116	70–110
Potassium (mmol/L)	3.3	3.5–5.0
Blood urea nitrogen (mg/dL)	14	7–18
Creatinine (mg/dL)	1.05	0.61–1.24
Total protein (g/dL)	8.6	6–8
Albumin (g/dL)	4	3.5–5.0

**Figure 1:**
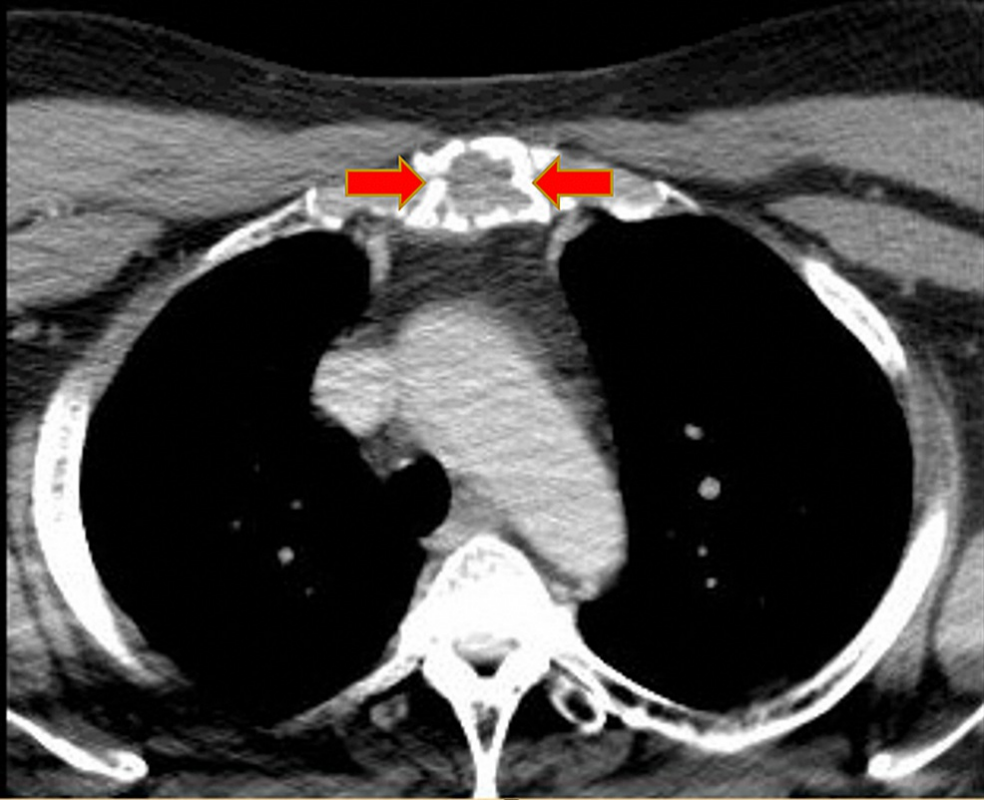
CT scan showing incompletely visualized expansile mass of the manubrium/upper sternum with irregularity and erosion of the cortex (red arrows)

**Figure 2:**
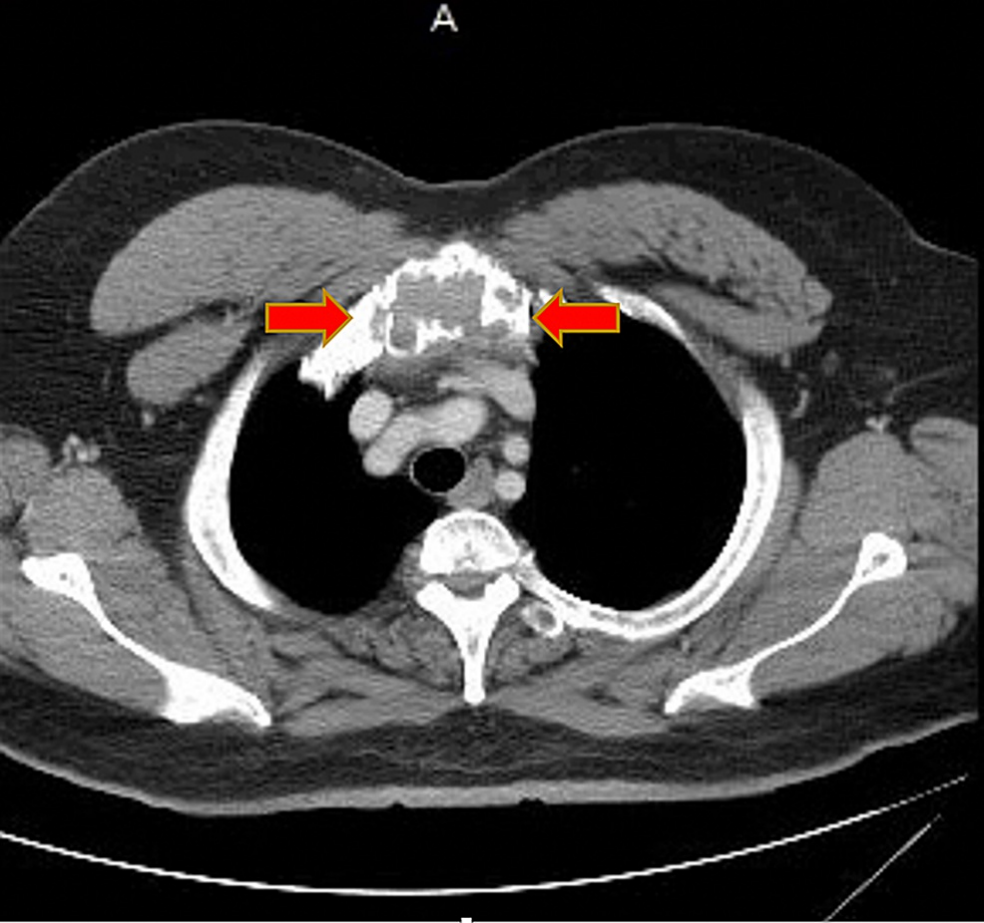
CT scan showing expansile, lytic lesion involving the manubrium (red arrows) A - anterior

**Figure 3:**
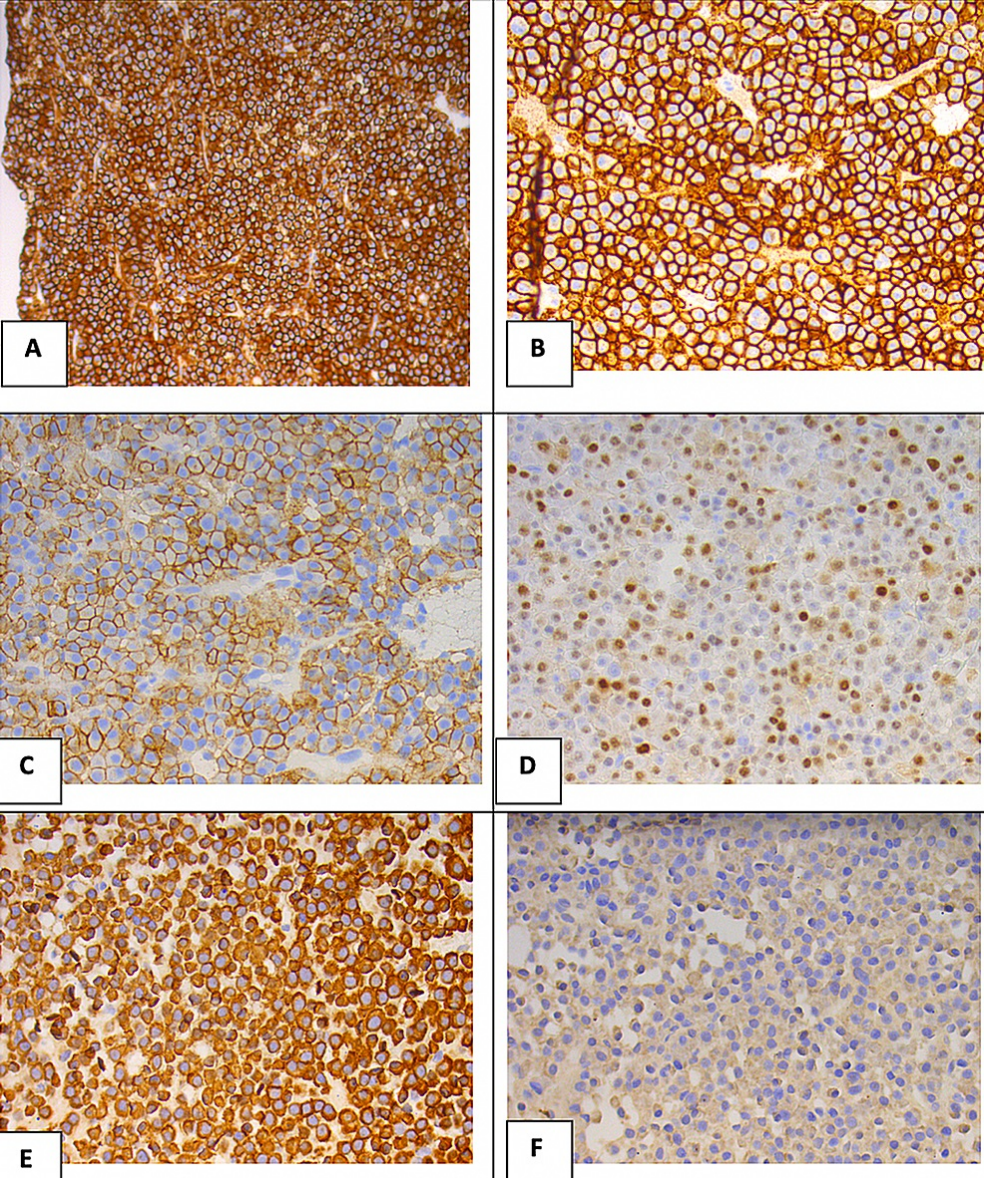
Histological features of myeloma cells with the following immunophenotypic profiles: (A) CD 138 (+), (B) CD 56 (+), (C) CD 117 (+), (D) cyclin D1 (+), (E) lambda-ISH (+), (F) kappa-ISH (-). CD - cluster differentiation; ISH - In situ hybridization

An electrocardiogram showed a normal sinus rhythm, with a rate of 80 beats per minute, and no ST-T-segment changes. Computed tomography (CT) soft tissue of the neck with and without contrast revealed an expansile mass within the manubrium, measuring about 2.4 x 4.8 cm in the greatest transaxial dimension and involving the entire visualized manubrium and upper sternum (Figure 1). The cortex looked somewhat lobulated and irregular with multiple defects within it. There was a high index of suspicion for metastatic disease or MM. CT of the chest with contrast revealed an expansile lytic lesion involving the manubrium (Figure 2). The CT of the head with contrast was unremarkable. Results from CT-guided manubrial mass core needle biopsy revealed histologic sections showing diffuse sheets of plasma cells with rare Dutcher bodies. These abnormal plasma cells showed the following immunophenotypic profile: positive cluster differentiation (CD) 138, 56, 117, cyclin D1 (subset), and lambda (Figures 3A-3F). These findings were consistent with a diagnosis of multiple myeloma. He was discharged home in stable condition with outpatient hematology and oncology follow-up.

**Figure 4:**
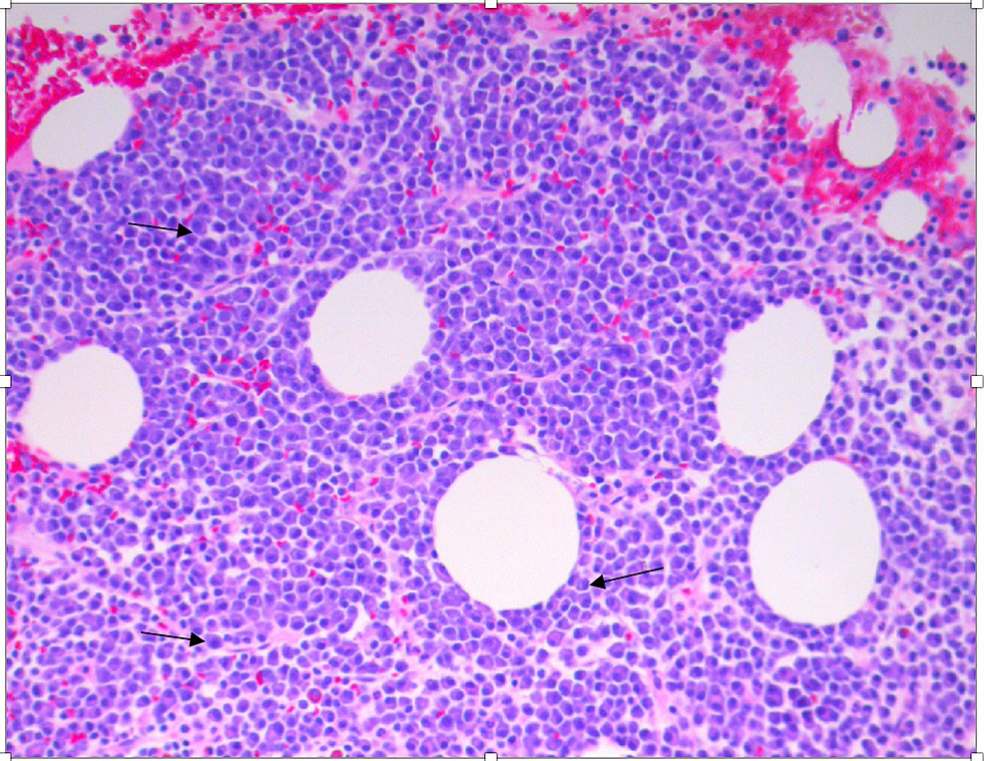
Histological feature of bone marrow biopsy, showing increased levels of atypical plasma cells

Thirteen days later, a scheduled right iliac crest bone marrow biopsy was performed using x-ray imaging. Results revealed an extensive interstitial infiltrate of atypical plasma cells, with rare plasma cells exhibiting immature nuclear features (binucleated/multinucleated). CD 138 stains showed variable infiltrates of atypical plasma cells, seen as large clusters and dense diffuse sheets accounting for approximately 60% of all marrow nucleated cells. Bone marrow smear revealed increased levels of atypical plasma cells with binucleation/multinucleation and vacuolated cytoplasm (Figure 4). All other cells (megakaryocytes and myeloid) were unremarkable, except mildly decreased erythroid cells (Table 2). Also, stored iron appeared mildly increased. With this confirmed diagnosis of MM, he was educated on the common symptoms and scheduled for an outpatient follow-up visit.

**Table 2:** Bone marrow differential cell count

Cell type	% of cells present	Normal adult range
Blasts	Rare	0–1
Promyelocytes	1	1–3
Myelocytes	1	2–10
Metamyelocytes	4	5–15
Bands	1	10–40
Neutrophils	6	10–30
Eosinophils	1	0–3
Basophils	Rare	0–1
Erythroid precursors	4	3–36
Monocyte	1	0–3
Lymphocyte	11	5–15
Plasma cells	70	0–3

## Discussion

MM is a rare plasma cell neoplasm with variable clinical manifestations. It involves the clonal infiltration of malignant plasma cells in the bone marrow. MM belongs to a family of monoclonal gammopathies; monoclonal gammopathy of undetermined significance (MGUS) and asymptomatic/smoldering myeloma accounting for about 1% of all cancers [1]. It is regarded as the most common primary bone cancer in older patients with a median age of onset of about 65 years [2,3]. In the United States, MM has an incidence of about 30,000 new cases per year, and in 2018, more than 10,000 people died of this disease [3].

According to the International Myeloma Working Group (IMWG), early diagnosis of MM requires the presence of ≥10% monoclonal plasma cells in the bone marrow or plasmacytoma, an increased ratio of serum-free light chains (kappa: lambda), and positive imaging findings [4]. Symptomatic features like bone pain, fatigue (anemia), kidney failure, and hypercalcemia help differentiate smoldering (asymptomatic) from symptomatic MM [2]. Although on physical examination there was a presence of a sternal hard mass in our patient, he complained of bone pain (manubrium) and bone marrow biopsy revealed 70% monoclonal plasma cells, which helped make the accurate diagnosis of symptomatic MM.

EM is a rare presentation that is defined as the accumulation of monoclonal plasma cells in an unusual position other than the bone marrow. It could be primary (initial diagnosis) or secondary (relapsing MM). The incidence of primary EM is about 4%-16% and the prognosis is believed to be poor [5]. In a cohort study published by Pour et al. in 2014, EM was grouped into three different types clinically: (i) abnormal infiltration of monoclonal cells in organs without any focal lesion, (ii) tumor mass not related/adjacent to bone, and (iii) bone-related tumor mass extending into soft tissues [4,5]. We believe that our patient has primary EM and also fits best with the last clinical classification. He presented with a soft tissue swelling (mass), which was adjacent to the sternal bone.

Pour et al. classified EM into two different prognostic groups; bone-related and non-bone-related EM. In this study, the median survival rate of patients with EM adjacent to bone was one year versus <5 months when compared to non-bone related EM from the onset of diagnosis [5]. Fortunately, we believe that due to the rarity of our patient’s presentation (chest pain), MM was diagnosed early and regular follow-up and aggressive treatment plans may help prolong his survival.

The detection of monoclonal protein (M-protein) in urine or blood also guides in determining the diagnosis of MM. A protein gap (total serum protein minus serum albumin) of >4 may be present in patients with monoclonal gammopathies. However, bone marrow biopsy is still required for definitive diagnosis of symptomatic MM versus MGUS and smoldering type [6]. This also helps providers risk-stratify patients who require urgent treatment from those who need closer follow-ups [6,7]. Magnetic resonance imaging is regarded to be most sensitive in detecting bone lesions in patients with this disease, but several other imaging tests, such as CT scans and skeletal x-ray surveys, could also aid in diagnosis [8]. Core needle biopsy is also required in patients with EM presenting with a tumor-like mass, like in our patient [8]. Our patient’s chest CT with contrast revealed lytic lesions and needle biopsy of the mass helped with early diagnosis of MM. The pathophysiology involved in the formation of EM as tumor-like mass is still not clearly explained or understood, and there is no definite treatment yet [6,9].

Despite the advancements in medicine, MM is still regarded as an incurable disease [6,10]. The management of MM is dependent on symptoms related to the disease process or complications. We believe that early detection of this disease may help increase the survival rate, and also limit those devastating complications associated with it. We recommend that older patients with bone pain in any location of the body should be screened for MM to decrease morbidity and mortality.

## Conclusions

EM is a rare clinical manifestation of MM. Due to its rarity, detailed history, physical examination, imaging studies, laboratory, and core-needle biopsy should be performed to aid appropriate diagnosis. Imaging, core needle, and bone marrow biopsy helped in diagnosing MM in our patient to initiate early treatment. Therefore, physicians should pay close attention to older patients with similar clinical presentations. Early detection of this devastating disease may help to increase the length of survival and also limit unexpected complications associated with it. 
